# Schwannoma of the Posterior Tibial Nerve Presenting as Tarsal Tunnel Syndrome: A Case Report with Emphasis on the Role of Microscope during Surgery

**DOI:** 10.1155/2018/4704362

**Published:** 2018-07-30

**Authors:** Raja Bhaskara Rajasekaran, Rajasekaran Shanmuganathan

**Affiliations:** Department of Orthopaedics & Trauma, Ganga Medical Centre & Hospitals Pvt. Ltd., 313 Mettupalayam Road, Coimbatore, India

## Abstract

Schwannoma is a benign, noninvasive tumour of the peripheral nerve sheath with rare occurrence in the extremities. We present a case of a schwannoma in the posterior tibial nerve which presented with symptoms suggestive of tarsal tunnel syndrome. The patient was managed with surgical excision of the tumour under microscope, and the diagnosis was confirmed by histopathology. Such a presentation is rare, and our case report adds light regarding the management of such cases.

## 1. Introduction

A schwannoma is a benign, encapsulated tumour which is noninvasive and derives its origin from Schwann cells. With no gender predilection, they typically occur between the ages of 20 and 50 years [1, 2]. They usually occur in the head and neck region, and a malignant transformation is very rare. They occur rarely in lower extremities and can mimic compression neuropathies at times [3]. We present a patient with features of tarsal tunnel syndrome diagnosed to be secondary to a schwannoma of the posterior tibial nerve. This is a rare presentation with minimal literature describing such conditions.

## 2. Case Report

**Figure 1:**
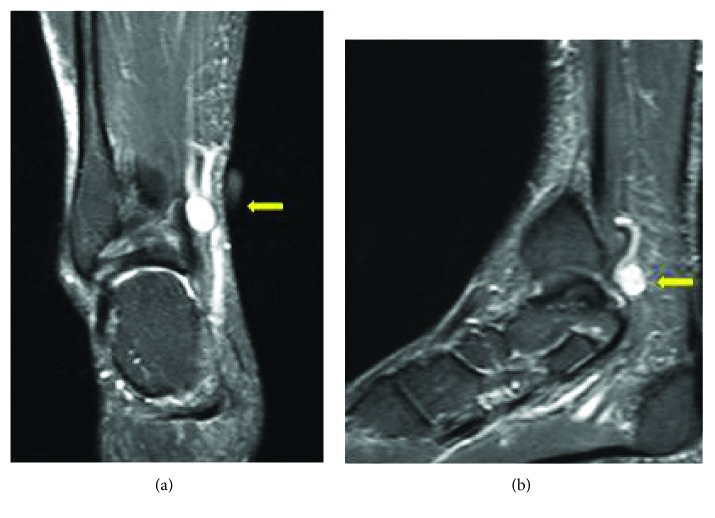
Well-defined hyperintense lesion (depicted by arrows) seen involving the posterior tibial nerve in T2-weighted MRI (a, b).

A 58-year-old banker presented to the outpatient department with complaints of pain over the medial aspect of the right foot associated with occasional numbness over the plantar aspect of the foot for a duration spanning 18 months. He had taken treatment outside for same condition and was misdiagnosed as lumbar radiculopathy. No mass was palpable on examination, but there was a positive Tinel sign along the course of the posterior tibial nerve. Motor and sensory examination of the foot were normal. Nerve conduction study showed an increased latency of the posterior tibial nerve with reduction in amplitude of motor unit. Magnetic resonance imaging (MRI) of the right ankle revealed a well-defined hyperintense lesion measuring 1.3 cm × 1 cm on T2-weighted images (Figures 1(a) and 1(b)). The lesion involved the posterior tibial nerve at the level of the ankle joint and involved a few nerve fibres medially. The lesion appeared to exert a mass effect on both the flexor hallucis longus and flexor digitorum longus tendons.

**Figure 2:**
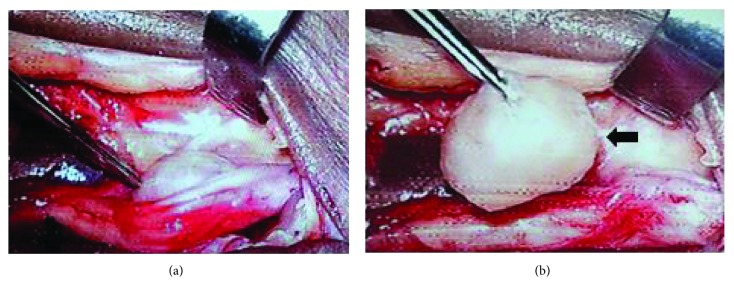
Intraoperative images (a, b) showing excision of the mass which was engulfed in the posterior tibial nerve as seen through the microscope which was used to assist excision.

Owing to the symptoms and findings on MRI, we planned to do a surgical excision of the tumour using microscope to aid in better visualization and finer dissection. Using a medial incision lateral to the posterior tibial artery, dissection of the tarsal tunnel was carried out. The posterior tibial nerve was identified, and there was a bulge noted along its course around the level of the ankle joint. Under microscope guidance, the nerve sheath was incised and a soft, ovoid mass with few fibres attached medially to the nerve sheath was noted (Figure 2(a)). Careful dissection of the mass was done (Figure 2(b)), and it was excised completely. The nerve fascicles were identified and left intact. Microscope use provided us with better magnification of the operating field and also helped in better dissection of the mass. The excised mass was 1.3 cm × 1 cm in diameter. Wound closure was done, and postoperatively, the patient had no sensory or motor weakness.

**Figure 3:**
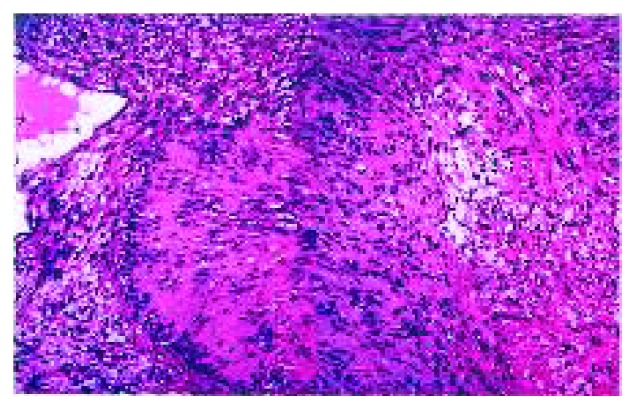
Histopathological examination revealing the presence of Verocay bodies with characteristic hypocelluar areas (Antoni A) with intermittent hypercellular areas (Antoni B) confirming the diagnosis of schwannoma.

Histopathological examination (Figure 3) showed the presence of characteristic hypocellular (Antoni A) areas with intermittent hypercellular (Antoni B) areas combined with the presence of Verocay bodies confirming the diagnosis of a benign schwannoma.

At a 6-month follow-up period after the surgery, the patient was completely free of pain with a VAS (Visual Analogue Score) score of 0 compared to the preoperative VAS score of 7, and there were no symptoms of numbness.

## 3. Discussion

Though schwannomas are the most common tumour of the peripheral nerve sheath, their occurrence in the lower extremities is limited to less than 10% [1–3]. Very few literature is present that shows a schwannoma in the tibial nerve to portray features of a compression neuropathy [4–9]. Albert et al. [3] in their series of three cases reported that each case had a varied presentation in comparison with the other. However, in all cases described, surgical excision gave good results and complete resolution of the symptoms.

Another aspect which is peculiar to these cases is the delay in diagnosis. Nawabi and Sinisi [4] in their series of 25 patients of a similar presentation showed that the mean time to diagnose the schwannoma was 86.5 months. We feel that the reason for delay of 18 months to diagnosis in our case was mainly due to two reasons similar to the ones described by Nawabi and Sinisi [4]. First, our patient had a lesion which was deep seated and was not palpable on routine examination. Secondly, a misdiagnosis of lumbar radiculopathy is common in cases of neuropathic pain around the foot in the absence of any obvious mass or pathology. These two factors can be overcome by careful examination where at times positive Tinel sign may be the only clue to diagnosis. Confirmation and localizing the lesion can be done using MRI as shown in our case. Ultrasound scan has also shown to be effective [10].

Once the diagnosis has been made, surgical excision is the treatment of choice [5, 6]. As seen in Table 1, we see that surgical excision has been followed in all similar cases giving good results. No recurrence is seen usually when a meticulous dissection has been done.

Based on our case and review of literature, we feel that any case of a neuropathy of the foot in the absence of any obvious evidence of lumbar radiculopathy or a compression neuropathy needs to be investigated further. In the early stages of the disease, a lump may not be palpable [4, 9]. Delay in diagnosis is a common feature in tumours of the peripheral nerves (Table 1) as they are usually deep seated or misdiagnosed. Here comes the importance of thorough clinical examination as in most cases, a positive Tinel sign along the course of the nerve may lead to the probable diagnosis which should be confirmed with an MRI.

Another important point to note is that the magnification during surgery is the key to achieve good outcomes as it helps with the dissection. Kim et al. [11] in their study showed that magnification is essential as it helps avoid damage to fascicles during excision. In their series, they had used loupe magnification to good effect whereas in our case, we used the microscope. Based on our experience, we suggest that the use of magnification assists the surgeon in better dissection and thorough excision of the tumour with minimal damage to the underlying nerve.

## 4. Conclusion

Our report shows that schwannoma of the posterior tibial nerve could present as tarsal tunnel syndrome, and a positive Tinel sign may be the only positive clinical sign to guide the clinician. Complete excision of the lesion by meticulous dissection protecting the nerve fascicles with the aid of a microscope or a loupe magnification could be the ideal way forward in managing such cases.

## Figures and Tables

**Table 1:** Tabulation of similar studies on schwannomas mimicking tarsal tunnel syndrome and comparing the delay in diagnosis, investigations performed, procedures done, use of magnification, and the recurrence.

Study	Delay in diagnosis	Investigations done	Procedure done	Use of magnification	Recurrence
Ghaly [9]	10 years	CT scan USG nuclear scintigraphy	Excision	No	Nil
Milnes and Pavier [5]	6 years	MRI	Excision	No	Nil
Kim et al. [11]	Mean of 3.42 years	MRI USG	Excision	Yes (loupe)	Nil
Nawabi and Sinisi [4]	Mean of 7.20 years	MRI in 80% NCV in 20%	Excision in all cases	No	Nil
Hallahan et al. [6]	Nil	MRI	Excision	No	Nil
Tladi et al. [7]	Mean of 12.5 years	MRI USG	Excision & neurolysis	No	Nil
Watanabe et al. [8]	2 years	MRI	Excision	No	Nil
Our case	1 and half year	MRI NCV	Excision	Yes (microscope)	Nil
